# Epidemiology of Thalassemia in Gulf Cooperation Council Countries: A Systematic Review

**DOI:** 10.1155/2020/1509501

**Published:** 2020-10-28

**Authors:** Amani Abu-Shaheen, Humariya Heena, Abdullah Nofal, Doaa A. Abdelmoety, Abdulrahman Almatary, Mohammed Alsheef, Isamme AlFayyad

**Affiliations:** ^1^Research Center, King Fahad Medical City, Riyadh, Saudi Arabia; ^2^Emergency Medicine Department, King Saud University Medical City, Riyadh, Saudi Arabia; ^3^Clinical Research Management Department, Executive Administration of Research, King Abdullah Medical City in Holy Capital, Makkah, Saudi Arabia; ^4^Neonatal Intensive Care Unit, King Fahad Medical City, Children Specialized Hospital, Riyadh, Saudi Arabia; ^5^Internal Medicine Consultant, King Fahad Medical City, Riyadh, Saudi Arabia

## Abstract

**Background:**

Thalassemia has a burden on the healthcare systems of many countries. About 56000 conceptions result in thalassemia, globally.

**Objective:**

To assess the epidemiological profile of thalassemia in the Gulf Cooperation Council (GCC) countries.

**Methods:**

A systematic search was conducted in MEDLINE/PubMed (National Library of Medicine), CINAHL, and Embase. Relevant observational studies reporting the epidemiology of thalassemia among the GCC population were selected. Data on the prevalence, frequency, and complications of thalassemia were extracted. The quality of the retrieved studies was assessed according to the Newcastle–Ottawa Quality Assessment Scale.

**Results:**

Eighteen studies (14 cross-sectional studies, two retrospective observational studies, and two retrospective analysis) with a total of 3343042 participants were included in this systematic review. Of the 18 studies, 11 studies were conducted in Saudi Arabia, two in the Kingdom of Bahrain, one in Kuwait, three in the United Arab Emirates (UAE), and one in Qatar. The prevalence of thalassemia among children below five years of age ranged from 0.25% to 33%, while it was 0.9% in children above five years and from 0.035% to 43.3% among adult thalassemia patients. The most-reported risk factors were consanguineous marriage and high-risk marriage. There was a lack of data regarding mortality rates in thalassemia.

**Conclusions:**

Despite the premarital screening and genetic counseling (PMSGC) program for thalassemia, the incidence of high-risk couple marriages in GCC countries cannot be effectively diminished. This study suggested that the PMSGC program should adopt more attention for the high-risk areas, to enhance the level of consciousness about the hemoglobinopathy diseases and the consequences of consanguinity among the at-risk couple.

## 1. Background

Thalassemia is a genetic blood disorder in which the body produces an abnormal form of hemoglobin. There are two types of thalassemia, depending on which type of globin is mutated: alpha- (*α*-) thalassemia and beta- (*β*-) thalassemia. *α*-Thalassemia occurs when one or more of the four *α*-globin genes are damaged or altered, while *β*-thalassemia occurs when both *β*-globin genes are damaged or mutated [1]. Furthermore, thalassemia major occurs when a child inherits two defective globin genes, one from each parent, and thalassemia minor occurs when the child inherits one defective globin gene from only one parent [2, 3]. One of the most-reported risk factors of thalassemia is consanguineous marriage. Consanguineous marriage is the marriage of two individuals having connections as second cousins with the inbreeding coefficient > 0 · 0156. The inbreeding coefficient is a method to measure the proportion of loci where the offspring of consanguineous marriage is predicted to receive identical gene copies from both parents [4, 5].

Patients with thalassemia minor show no manifestations and can have a healthy life without treatment, whereas patients with thalassemia major usually experience lifelong anemia that begins in early childhood, and due to the abnormality of the red blood cells, the patient must be managed with regular blood transfusions [1]. Homozygous *α*-thalassemia occurs due to the deletion of all four *α*-globin genes. *β*-Thalassemia phenotypes are heterogeneous, varying between severe transfusion-dependent thalassemia major to the mild thalassemia intermedia form. Patients with *β*-thalassemia major develop severe anemia and hepatosplenomegaly, and the children with this condition, if not treated, show developmental delay and reduced life expectancy [3]. Previously *α*-thalassemia disorders were thought to be benign; however, homozygous *α*-thalassemia has been found currently to be fatal and commonly detected in populations worldwide [1, 6]. Homozygous *α*-thalassemia results in Hb Barts formation in utero. Due to the extremely high affinity of Hb Barts, little oxygen is delivered to fetal tissues causing severe hypoxia, cardiac failure, and intrauterine death of the fetus [6, 7]. In the case of homozygous *α*-thalassemia, early detection followed by intrauterine transfusion can prevent fetal death, but the transfusion therapy requires to be continued [6]. The management of thalassemia requires high bloodstock, which highly consumes blood at the expense of other treatments that requires a blood transfusion, and it causes significant complications such as iron overload, bone distortions, and cardiovascular disorders due to lifelong blood transfusion [6, 7].

About 56000 conceptions result in thalassemia, globally. Of them, approximately 30000 are affected by *β*-thalassemia, and around 3500 succumb perinatally from the hydrops fetalis or *α*-thalassemia syndrome [1]. Moreover, it was estimated that annually, about nine million thalassemia carrier women around the world get pregnant, and of them, 1.33 million pregnancies are at risk for major thalassemia disorder [1, 8, 9]. Thalassemia has a burden on the healthcare systems of many countries in the Mediterranean area, the Middle Eastern and North African areas (MENA), Central Asia, Transcaucasia, the Indian subcontinent, and Southeast Asia [6, 7]. The *α*-thalassemia gene frequencies are found to be higher than *β*-thalassemia, and in the regions in Southeast Asia, the frequency of *α*-thalassemia reached 25% [6, 7]. On the other hand, the countries in the MENA region, particularly UAE and other Gulf Cooperation Council (GCC) countries, the prevalence of *β*-thalassemia major and carriers is higher than *α*-thalassemia [1].

Many countries have measured the knowledge and the attitudes of the public towards thalassemia to implement and evaluate relevant educational programs [10, 11]. Assessment of the population's awareness of *β*-thalassemia documented that there is a need to improve the population's basic knowledge of the disease [11]. The perception of the public about thalassemia was found to be associated with several factors, including gender, marital status, education, employment, and socioeconomic status, and these factors vary by region and community [12, 13].

In developing countries, many thalassemic patients die because of expensive management and the absence of adequate measures, which appears to restrict early thalassemia symptoms [14–17]. Due to the therapeutic advances, the major thalassemia patients experienced an increased life expectancy, and the disease changed from a deadly disorder to chronic disease with a need for lifetime care [18–20]. The lifetime care of thalassemia major patient is costly, and it includes the cost of (1) blood transfusion and iron chelation medications, (2) laboratory investigations, (3) management of adverse events, (4) recurrent visits, and (5) indirect expenses such as the costs of missed chances and quality of life [16, 21, 22].

Some studies were conducted to assess the management of thalassemia expenses. In the United Kingdom, Karnon et al. stated that the discounted lifetime cost of managing a *β*-thalassemia major patient was estimated to be 188000 to 226000 pounds [23]. While in Thailand, Riewpaiboon et al. recruited 201 patients and documented that the yearly average management cost was 950 United States dollars (US$), among which 59% was direct medical cost, 17% direct nonmedical cost, and 24% indirect cost [24].

This article is aimed at systemically reviewing the epidemiological profile represented by the frequency, prevalence, risk factors, mortality rates, and complications of thalassemia in GCC countries that will help in the development of a consensus on newborn screening programs and strategies that may reduce the mortality of newborn due to thalassemia. Moreover, it will help in improving the clinical outcomes and quality of life for thalassemia patients.

## 2. Methods

This systematic review was based on the Preferred Reporting Items for Systematic Review and Meta-analysis (PRISMA) statement [25], and all steps were performed according to the Cochrane Handbook [26].

### 2.1. Literature Search Strategy

An electronic database search was conducted through MEDLINE/PubMed (National Library of Medicine), CINAHL, and Embase for relevant published studies from the beginning until December 31, 2018. The leading search criterion was the appearance of the term thalassemia in GCC countries. We combined other terms of hemoglobinopathies, such as sickle cell trait, sickle cell disease, and sickle cell hemoglobin, so that we did not miss any study, including thalassemia. The search strategy is shown in the supplementary (S1) file.

### 2.2. Eligibility Criteria

#### 2.2.1. Inclusion Criteria

Observational studies on population diagnosed with thalassemia in GCC countries and studies that reported the epidemiology profile of thalassemia regarding the frequency, prevalence, risk factors, mortality rates, and complications were included. No restrictions for language were applied.

#### 2.2.2. Exclusion Criteria

Laboratory diagnostic tests, experimental and animal studies, studies conducted in other countries, review articles, case reports and case series, and conference proceedings and editorials were excluded. Moreover, the studies not relevant to the research aim or not providing necessary data on the epidemiological profile of thalassemia were not included in this systematic review.

### 2.3. Study Selection and Data Extraction

Eligibility screening was conducted in two steps: (a) title and abstract screening for matching the inclusion criteria and (b) full-text screening of selected articles retrieved for comprehensive analysis and further assessed for final inclusion. Two researchers (AKA and INF) independently reviewed each study, and disagreement was resolved by consensus following a discussion between the two reviewers.

### 2.4. Assessment of Risk of Bias

The quality of the retrieved studies was assessed according to the Newcastle–Ottawa Quality Assessment Scale (NOQAS) developed for cohort studies [27], and the modified version of the NOQAS for cross-sectional studies [28]. The NOQAS is a valid instrument that assigns a maximum of four points for selection, two points for comparability, and three points for exposure or outcome. The NOQAS score of 7 was considered the cut-off for a high-quality study methodology and a score of 5–6 as moderate quality [29]. Quality assessment was conducted by two independent reviewers.

### 2.5. Data Extraction

We independently extracted and verified the data from each included study. Extracted data included the following domains: first author name, year and location of publication, study period, study design, participants' characteristics, sample size, country, and primary outcome measures (frequency, prevalence, and complications).

## 3. Results

**Figure 1:**
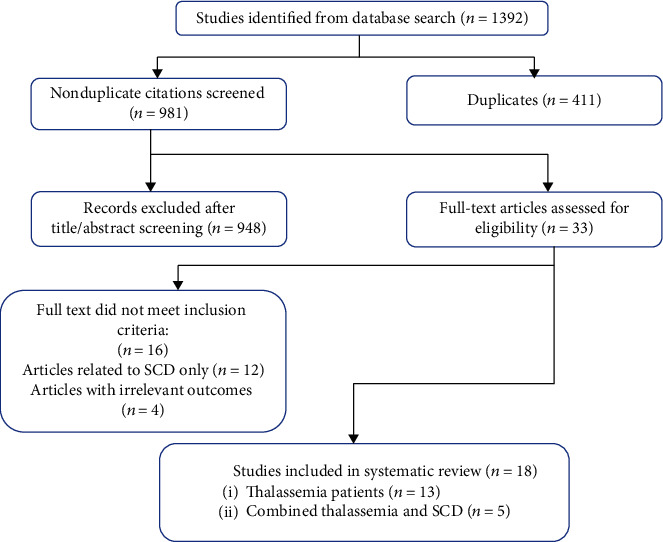
Flow diagram of the literature search and study selection.

The literature search yielded 981 nonduplicate citations, following title and abstract screening; 33 articles were eligible for full-text screening and were examined in detail. Finally, 18 articles [30–47] were included in our systematic review. The screening process is shown in Figure 1.

A total of 3343042 participants were included in this systematic review. The patients were GCC population diagnosed with thalassemia (either *α* or *β* type). The retrieved articles included 14 cross-sectional studies, two retrospective observational studies, and two retrospective analyses. Of the 18 studies, 11 studies [30–32, 39–46] were conducted in Saudi Arabia, two studies in the Kingdom of Bahrain [33, 34], one study in Kuwait [35], three studies in the United Arab Emirates (UAE) [36–38], and one study in Qatar [47]. The summary of the baseline characteristics of the study populations is shown in Table 1.

Eleven studies were of fair quality in methodology [30, 31, 33, 34, 37–39, 41, 42, 45, 47], two studies were of good quality, [35, 36] two studies were of poor quality [40, 46], and the quality of three studies was not quite clear. [32, 43, 44] Table 2 provided the quality scores of the included studies according to NOQAS criteria.

As shown in Table 3, sixteen studies reported the prevalence of thalassemia in GCC countries; of them, nine studies [30–32, 39, 41, 42, 44–46] were conducted in Saudi Arabia, and the remaining seven studies [33–38, 47] were conducted in UAE, Kingdom of Bahrain, Kuwait, and Qatar. We determined the prevalence of thalassemia as the proportion of a particular population being affected by thalassemia.

The age groups of the study populations varied, including children below five years of age, children above five years, and adults (Table 3). Only one study [44] in Saudi Arabia included participants of all three age groups, and the overall prevalence of thalassemia was found to be 30.72% in this study. Other studies included participants of any of the two age groups or exclusively one age group. Three studies [31, 38, 46] (two in Saudi Arabia and one in UAE) were conducted only on the children below five years with the prevalence of *α*-thalassemia ranging between 36% and 49% and 0.25% for diseased *β*-thalassemia (6 out of 2341). Both the lowest and highest prevalence of thalassemia were detected in Saudi Arabia among the examined population. Perrine et al. [31] in a study with 2341 infants detected diseased *β*-thalassemia with sickle cell disease in six infants (0.25%). Nasserullah et al. [46] in a study in Saudi Arabia screened the blood of 21858 newborns and detected *α*-thalassemia (carrier or diseased) in 7923 newborns (36%). A study by Barakat-Haddad et al. [36] in UAE was conducted only on the children above five years (adolescent students age 15-18 years) with the reported thalassemia (carriers or diseased) prevalence of 0.9%. Seven studies [32, 33, 37, 39, 41, 45, 47] were conducted solely on adult thalassemia (carrier or diseased) patients, with the prevalence of overall thalassemia (carrier or diseased) ranging from 0.035% to 43.3%. The lowest prevalence of diseased *β*-thalassemia (0.035%; 2 out of 5672 individuals) was reported by Denic et al. [37] in UAE and the highest prevalence of *β*-thalassemia 28.9% was reported by Fawzi et al. Ganeshaguru et al. [32] also reported the prevalence of *α*-/*β*-thalassemia and *α*-thalassemia to be 0.9%, and 43.3%, respectively. Furthermore, Denic et al. [37] included adults with a total number of 5672 and documented that the *β*-thalassemia allele frequency was 1·16%. El-Kalla et al. [38] in their study included 418 children below five years to analyze *α*-globin gene status and found that 11% (46/418) were homozygous for *α*-gene deletion. Rajab et al. [33] conducted a study in Bahrain with pregnant women having sickle cell disease and found that 9.6% had sickle cell hemoglobin with *β*-thalassemia disease. Fawzi et al. conducted a study in Qatar with 3275 adults and detected that 1.37% (includes Qatari and non-Qatari patients) and 0.76% (Qatari patients) of the patients were *β*-thalassemia major [47].

Consanguineous marriage and high-risk marriage (in which two couples have genetic defects) are the most reported risk factors of thalassemia. Alsaeed et al. [42], AlHamdan et al. [41], and Memish et al. [45] reported consanguineous marriage to be an important risk factor on the incidence of thalassemia in Saudi Arabia. Moreover, Denic et al. [37] reported data about high-risk marriage and its impact on the incidence of thalassemia in UAE.

Three studies [30, 40, 43] documented occurrence of splenic and hepatic complications among thalassemia patients in Saudi Arabia. Ankra-badu et al. [43] recorded 56% of *α*-thalassemia patients had splenomegaly with the size of the spleen ranged from 1 to 13 cm, 41% of the patients had hepatomegaly, and 35·9% had jaundice. El-Hazmi et al. [40] reported that 14% of the included *α*-thalassemia patients experienced splenomegaly, and 14% of patients had jaundice.

Hematological complication, resulting in blood transfusion, was reported by three studies in Saudi Arabia [30, 40, 43]. Ankra-badu et al. [43] reported 26 out of 36 *α*-thalassemia patients (72·2%) had blood transfusion, while El-Hazmi et al. [40] reported blood transfusion in 30% *α*-thalassemia patients, and Pembrey et al. [30] reported blood transfusion in four out of 16 *β*-thalassaemia patients (25%). Likewise, anemia was reported to occur in 30% and 56.4% of the included participants in Saudi Arabia by El-Hazmi et al. [40] and Ankra-badu et al. [43], respectively.

Two studies [30, 40] reported various types of infections, such as upper and lower respiratory tract infections and osteomyelitis in Saudi Arabia. Pembrey et al. [30] reported that four out of 16 *β*-thalassemia patients had upper and lower respiratory tract infections, while El-Hazmi et al. (1985) [40] documented that 30% of *α*-thalassemia patients experienced osteomyelitis.

There was a lack of data regarding mortality rates of thalassemia in the included studies.

## 4. Discussion

The objective of this systematic review was to assess the epidemiology of thalassemia in GCC countries. The prevalence of *β*-thalassemia (carrier or diseased) in Saudi Arabia is considered to be one of the highest rates in comparison to other Middle Eastern countries. [42, 45] The overall prevalence of thalassemia (carrier or diseased) rates ranged from 0.035% to 43.3% for adults with the lowest prevalence in the UAE, reported by Denic et al. [37] and the highest prevalence in Saudi Arabia, reported by Ganeshaguru et al. [32]. Similarly, for children below five years, the prevalence of *α*-thalassemia (carrier or diseased) ranged from 36% to 49% with the highest prevalence reported by Nasserullah et al. [46] and the lowest prevalence of diseased *β*-thalassemia (0.25%) reported by Perrine et al. [31] in Saudi Arabia. For children above five years, the reported thalassemia (carrier or diseased) prevalence was 0.9% [36]. According to Alsaeed et al., [42] the prevalence of diseased *β*-thalassemia was higher in the Western region of Saudi Arabia (1.9% in Makkah) than the Eastern region (0.4%) during the period 2011–2015, whereas AlHamdan et al. [41] reported a lower prevalence of diseased *β*-thalassemia in Makkah (0.01%) for adults screened during the Saudi Premarital Screening Program (February 2004 to January 2005). However, both the studies [41, 42] showed a high prevalence of *β*-thalassemia (carriers) in Jazan and Eastern Saudi Arabia. In the UAE, the overall prevalence of thalassemia (carriers or diseased) ranged between 0.035% and 11%. In Kuwait, the prevalence of diseased thalassemia was 30.7%, and in Bahrain, the prevalence of diseased thalassemia ranged between 0.09 and 9.6%, respectively. In Qatar, the prevalence of *β*-thalassemia major was 1.37% (includes Qatari and Non-Qatari patients) and 0.76% (Qatari patients). Moreover, compared to *α*-thalassemia (carrier or diseased), *β*-thalassemia (carrier or diseased) occurred more commonly in Qatar with *β*-thalassemia accounting for 71.25% of all thalassemias and *α*-thalassemia (carrier or diseased) accounting for only 28.75% of all thalassemias [47]. The cooccurrence of *β*-thalassemia (carrier or diseased) and sickle cell disease among the study participants was observed in Saudi Arabia (11.43%, 0.25%, and 3.3%) [30, 31, 39], Bahrain (12%) [33], and Qatar (2.05%) [47].

Kim et al. [1] estimated the prevalence of thalassemia major and carrier populations in the Middle East and the North African (MENA) region, as these regions show a high rate of consanguineous marriage (the principal risk factor for thalassemia major) and stated that Saudi Arabia had a high prevalence of *β*-thalassemia carriers (1–15%) and *α*-thalassemia carriers (5–10%). In Jordan, the prevalence ranged from 3% to 5·9% for *β*-thalassemia carriers and from 2% to 3·5% for *α*-thalassemia carriers [1]. In Egypt, the frequency of *β*-thalassemia carriers was 4.5%, and in Kuwait, the frequency of *α*-thalassemia carriers ranged from 5% to 10%. UAE showed a higher prevalence of both *β* and *α*-thalassemia carriers than Bahrain with the prevalence of *β*-thalassemia as 8·5% vs. 2·9% and *α*-thalassemia as 49% vs. 24·2% vs. [1].

According to a study by Modell and Darlison, the estimated rate of diseased thalassemia in affected conceptions (per 1000) among the American population (853 million) was 0·06%, among the African population (586 million) was 0·07%, among the European population (879 million) was 0.13%, among Southeast Asian population (1564 million) was 0.66%, and in the Western Pacific population (1.761 million) was 0·76%. The estimated prevalence of thalassemia in affected conceptions worldwide (6217 million) is 0·46% [9].

A study by Vichinsky et al. [48] in North America stated that *β*-thalassemia (carrier or diseased) predominated in Eastern North America and *α*-thalassemia (carrier or diseased) in the Western part of North America. Moreover, this study showed that the epidemiology of thalassemia in North America had wide heterogeneity. Hickman et al. [49] stated that in England, approximately 2800 babies were born with *β*-thalassemia trait annually, and among them, about 43 or 0.07 per 1000 conceptions were affected by *β*-thalassemia. A prospective cohort study in Ontario, Canada, by Lafferty et al. (2007) [50] reported 34.8% of the study population to be the carrier of thalassemia with a 24.5% probability of having children with *β*-thalassemia major.

*β*-Thalassemia major leads to intense anemia that can even cause the death of untreated children aged 3 years or less. Regular blood transfusion can prevent death due to thalassemia major [9]. In Saudi Arabia, blood transfusion among the included patients was reported as 72.2%, 30%, and 25% by Ankra-badu et al. [43], El-Hazmi et al. (1982) [44], and Pembrey et al. (1980) [30], respectively. However, most transfused patients usually suffer from iron overload, which may lead to death, and this can be overcome by using iron-chelation therapy.

Thalassemia requires lifelong treatment, including blood transfusion, use of iron-chelating agents, and other medications, which is expensive. Also, there are some other costs, such as medical consultation fees, diagnostic and laboratory tests, and treatment of adverse effects of therapies. Since most children in thalassemia are born in low-income countries, the high cost of therapy leads to a huge economic burden on the patient's family and society [9, 22]. Moreover, the rising cost of treatment is a major challenge for the health care funders and the government, and thus, the entire healthcare system of a country is affected [22]. Iron-chelation drug contributes significantly to the treatment expenses. Therefore, the economic burden of thalassemia on the health care system may be averted, by an effective, inexpensive oral iron chelator [51] so that the lifespan of patients receiving a periodic blood transfusion and iron-chelation medication can be extended [9].

Another effective means to reduce the occurrence of thalassemia is the preventive approach, which is followed in many countries to lower the financial burden [9]. It is evident that prevention is cheaper than treatment in the long term approach [22]. The preventive approach includes the following steps: (i) detecting the carriers of the disease and informing them about the risk of having children with thalassemia and the ways of lowering it, which would result in the reduction of births and deaths of affected children; (ii) prenatal diagnosis of couples with affected children and informing them about 25% recurrence risk and thus to limit family size, which may reduce the birth prevalence; and (iii) prospective carrier screening for the entire population. In countries with a high rate of consanguineous marriage, population screening is useful due to cost-effectiveness. The screening can be started among the high-risk couples before marriage. Screening of the carrier via prenatal diagnosis is being practiced in China, Hong Kong, some parts of India and Taiwan, Iran, Singapore, and most of southern Europe. In North America, Australia, New Zealand, and Northwest Europe, in addition to prenatal diagnosis, antenatal carrier screening is typically practiced. A global estimate indicated a 16% reduction in the birth of children with thalassemia by following the preventive approach. The education of at-risk couples resulted in a higher reduction of reproduction compared to prenatal diagnosis [9].

Consequently, Saudi Arabia launched a national obligatory premarital examination screening (PMSGC) program for thalassemia, which was introduced in 2004 in all regions of the country at free of cost. [52, 53] Hamamy et al. reported the frequency of carriers of *α*-thalassemia and *β*-thalassemia in Arab countries ranged from 1% to 58% and from 1% to 11%, respectively, having the highest frequencies in Gulf countries [54]. Therefore, implementation of public health approaches to prevent hemoglobinopathies in Arab countries, including newborn screening and premarital screening for carriers of thalassemia, should be improved [9, 54].

Although the testing is obligatory, the decision of the high-risk cases whether to proceed with the marriage or not depends on the couple [52]. Despite genetic counseling, the premarital testing program in Saudi Arabia has not effectively decreased the incidence of high-risk couple marriages, as the percentage of the high-risk couple who proceeded with their marriage was 90% in 2007 and 98% in 2010 [41, 55]. In 2014, the Saudi Ministry of Health indicated that 3000 out of 7500 individuals who were “physically incompatible” for thalassemia against medical advice were married [53].

The UAE has a 50% consanguineous marriage rate and a considerable prevalence of thalassemia carriers [56, 57]. This high prevalence indicates a public health concern since there will be a 25% chance of having a child with thalassemia major if a carrier-carrier marriage occurs, leading to a likely increase in thalassemia major population; moreover, the UAE has the most varied heterozygotes of *β*-thalassemia [56, 58].

In 2008, the UAE government introduced a nationwide campaign to promote premarital screening, which provides a nondirective genetic counseling to at-risk couples [59]. At-risk couples obtain information about the risk of having a child with thalassemia and decisions of prenatal and neonatal diagnosis. The final decision to proceed or to decline marriage depends on them, and many at-risk couples choose to proceed with the marriage [59]. The termination of pregnancy is not practiced as a solution for the prevention of thalassemia in the UAE [1]. However, the number of affected births is halved in comparison with the time before the introduction of the preventive screening program [1].

Two strategies have been made to manage thalassemia; the first strategy was the premarital screening to prevent the carrier population intermarriage, and the second strategy was educating young adults about thalassemia to decline marriage among the carrier populations. The baseline expectation was the reduction of the major thalassemia population and the eventual elimination of thalassemia.

As in many other countries, UAE also celebrates a free rank of thalassemia due to various prevention methods [60]. Since a high proportion of the working population resides in this relatively young country, the number of thalassemia carriers will simultaneously increase with the growing UAE population [1]. This predicts that in the UAE, the prevalence of thalassemia among the at-risk population will be enhanced by marriage among the carrier population, and thalassemia cases are highly likely to reappear.

In Oman, Hassan et al. documented that thalassemia was one of the main problems faced by public health in Oman, and untreated thalassemia patients are likely to die early in infancy and treatment is required to delay the premature death [61]. Options offered to prevent the disease, such as premarital screening and genetic counseling of high-risk couples, are limited to partner choice as the prenatal diagnosis, and medical abortion of the affected fetus is not permitted in Oman. Moreover, there is no clear distribution of thalassemia in Kuwait [62].

In Kuwait, Adekile et al. [62] studied the globin gene mutations associated with *β*-thalassemia intermedia and concluded that screening should commence with these two common alleles in Kuwaiti patients presenting with *β*-thalassemia syndrome. The early examination will avoid the complications of an additional hyper transfusion program.

AlHamdan et al. conducted a cross-sectional study in Saudi Arabia and estimated the prevalence of thalassemia (carrier or diseased) to be 0.07% of the screened 488315 individuals. Thalassemia was found to be focused mainly in the eastern, western, and southwestern parts of the country, indicating the need for improvement in public health education [41]. A study conducted by Al Jaouni et al. screened 7584 candidates and reported the prevalence of *α*-thalassemia to be 40.0% in Jeddah and concluded that the Saudi population in Jeddah is at risk for hemoglobin disorders. Screening procedures are essential and should be implemented in the prevention program as a routine practice [63].

In Bahrain, Hajeri et al. performed a cross-sectional survey to assess the public knowledge of *β*-thalassemia and found that 1297 individuals (65.1%) knew about *β*-thalassemia, 809 individuals (40·5%) knew that both parents must be carriers to inherit the disease to a child, and 1547 individuals (77·8%) agreed that premarital checking could prevent *β*-thalassemia. Females were found to have more knowledge than males. Also, married individuals seem to know more about *β*-thalassemia than the unmarried ones [11].

A study was conducted by Hassan et al. [61] in Oman to determine the frequency and distribution of the various *β*-alleles. The study examined 446 cases from seven different regions with *β*-hemoglobinopathies and concluded that the wide heterogeneous spectrum of *β*-thalassemia mutations could be associated with the history of trade and migration.

Similarly, Kamal et al. in Qatar examined the association between microcytic anemia and *α*-thalassemia in children. The study reported that a significant number of the pediatric population with microcytic hypochromic anemia selected through preschool health assessment programs at public schools are carriers of *α*-thalassemia mutations [64].

In UAE, Kim et al. [1] demonstrated whether thalassemia prevention methods such as premarital screening and education can result in the eradication of thalassemia in the long term via a mathematical model by studying different subgroups among the population, such as three age groups, three genetic groups, two screening groups, and two thalassemia education groups. The authors concluded that these factors could decrease the prevalence of the disease only for the short term but not eradicate the condition in the long-term period [1]. Moreover, Attia et al. screened 17826 individuals in the UAE and showed that the prevalence of *β*-thalassemia among the total study population was 2.98%, and 28 (0,31%) couples were declared high-risk hemoglobinopathy [65].

Olwi et al. [53] had conducted a cross-sectional survey of a random sample of 920 senior students at King Abdulaziz University, Saudi Arabia. A questionnaire was used to collect data about thalassemia and sociodemographic characteristics. From this survey, they observed that Saudi Arabia has one of the highest prevalence of thalassemia, and the country's effort to reduce the rate of thalassemia through premarital testing has not significantly decreased the rate of marriage among high-risk couples.

This systematic review has several strengths. The search strategy was quite comprehensive using many electronic databases, and a large number of a good quality of studies were included in this review, and only two studies out of the 18 studies were of poor quality.

However, the main limitation of this systematic review was the lack of data from other GCC countries, and the data reporting the mortality rates and the possible complications.

## 5. Conclusion

Despite the national obligatory premarital examination screening (PMSGC) program for thalassemia, the premarital testing program could not effectively diminish the incidence of high-risk couple marriages. However, from the previous study findings, it can be recommended that targeting young adults and increasing their awareness about thalassemia and its burden would decline marriage among the carrier populations and decrease the incidence of such disorders.

## Figures and Tables

**Table 1:** Characteristics of the included studies (*n* = 3339767 participants).

Country	Study ID	Disease	Age of sample	Children below five years	Children above five years	Adults	Location of publication	Study period	Study design	Participant characteristics	Sample size
Patients with thalassemia											
Saudi Arabia	AlHamdan et al. (2007) [41]	*β*-Thalassemia	NA	No	No	Yes	American College of Medical Genetics	Two years	Cross-sectional study	Individuals who applied for a marriage license	488315
	Alsaeed et al. (2011) [39]	*β*-Thalassemia	Mean (SD) = 39 (6.4)	No	No	Yes	Genetic Testing and Molecular Biomarkers	Five years	Cross-sectional study	Three hundred and twenty-nine (329) blood samples from suspected cases of both sexes (140 men and 189 women)	120
	Alsaeed et al. (2018) [42]	*β*-Thalassemia	Range 13-112 years for female and 17-105 for males	No	Yes	Yes	Journal of Epidemiology and Global Health	Five years	Cross-sectional study	Individuals attended the premarital screening centers distributed across the 13 administrative regions in Saudi Arabia	1230582
	Ankra-badu et al. (2001) [43]	*α*-Thalassemia	Mean (SD) = 18.0 (11.5)	Yes	Yes	Yes	Annals of Saudi Medicine	Two years	Retrospective analysis	The hematology department patients at Qatif Central Hospital were found to have low red cell indices during evaluation. There were 24 female (61.5%) and 15 male (38.5%) patients. Thirty-eight (38) of the patients (97.4%) were from the eastern province of Saudi, and all but three came from the Al-Qatif area. The only non-Saudi patient came from Bahrain	39
	El-Hazmi et al. (1982) [44]	Thalassemia	NA	Yes	Yes	Yes	Acta Haematologica	NA	Cross-sectional study	Students of local schools and/or outpatient clinics of hospitals from different regions of the Arabian peninsula	2643
	Memish et al. (2011) [45]	*β*-Thalassemia	NA	No	No	Yes	Annals of Saudi Medicine	Six years	Cross-sectional study	Saudi couples with marriage proposals between 2004 and 2009	1572140
	Nasserullah et al. (2003) [46]	*α*-Thalassemia	NA	Yes	No	No	Annals of Saudi Medicine	Nine years	Cross-sectional study	Newborns in Qatif Central Hospital 21858 (91.03%) were Saudi, and 2154 (8.97%) were non-Saudi had cord blood screening for sickle cell disease and other hemoglobinopathies	24012
	Ganeshaguru et al. (1987) [32]	*α*- and *β*-thalassemia	NA	No	No	Yes	Tropical and Geographical Medicine	NA	Cross-sectional study	Healthy adult male Saudi Arabians of different tribal origins	840
United Arab Emirates (UAE)	Barakat-Haddad et al. (2013) [36]	Thalassemia	Mean (SD) = 16.2 (1.2)	No	Yes	No	Journal of Environmental and Public Health	Two years	Cross-sectional study	Adolescent students who reside in the United Arab Emirates (UAE)	6329
	Denic et al. (2013) [37]	*β*-Thalassemia	Mean = 27.3 years (range 13 − 82)	No	Yes	Yes	Hemoglobin	Eight months	Cross-sectional study	Subjects who were going to marry in eight centers in Abu Dhabi, UAE. Individuals were screened for *β*-thalassemia, 3045 (53.5%) males and 2627 (46.7%) females	5672
	El-Kalla et al. (1998) [38]	*α*-Thalassemia	NA	Yes	No	No	Acta Haematologica	NA	Cross-sectional study	Newborns of UAE nationality	418
Kingdom of Bahrain	Al-Arrayed et al. (2003) [34]	*β*-Thalassemia	Range = 16–20 years	No	Yes	Yes	Eastern Mediterranean Health Journal	Ten months	Cross-sectional study	Bahraini and non-Bahraini students between 16 and 17 years old underwent screening for hemoglobinopathies	5685
Kuwait	Adekile et al. (1996) [35]	*α*-Thalassemia	Mean (SD) = 8.0 (6.1)	Yes	Yes	No	Acta Haematologica	2.5 years	Retrospective study	Patients records in the pediatric wards of the two major teaching hospitals in Kuwait (Mubarak Al-Kabeer and Al-Amiri) and all sickle cell anemia patients referred for follow-up in the pediatric hematology clinics of the two hospitals. 21 males and 18 females	39
Patients with *β*-thalassemia and sickle cell disease											
Saudi Arabia	Pembrey et al. (1980) [30]	Sickle cell disease + *β*-thalassemia	Range = (6 months − 16 years)	Yes	Yes	No	American Journal of Human Genetics	NA	Cross-sectional study	Patients aged between 6 months and 16 years attending the medical department of the Arabian American Oil Company (ARAMCO)	140
	Perrine et al. (1981) [31]	Sickle cell disease + *β*-thalassemia	NA	Yes	No	No	Archives of Disease in Childhood	NA	Cross-sectional study	Infants of the employees of the ARAMCO from the oases of Eastern Saudi Arabia	2341
	Alsaeed et al. (2011) [39]	*β*-Thalassemia	Mean (SD) = 39 (6.4)	No	No	Yes	Genetic Testing and Molecular Biomarkers	Five years	Cross-sectional study	Three hundred and twenty-nine (329) blood samples from suspected cases of both sexes (140 men and 189 women)	11
	El-hazmi et al. (1985) [40]	Sickle cell disease + *β*-thalassemia	Mean age = 17 years, range (5 to 50 years)	No	Yes	Yes	Acta Haematologica	NA	Retrospective analysis	Saudi patients with 1 or 2 *α*-gene deletions. Samples were collected during field trips to Al-Hofuf, Al-Qateef, Dammam, and neighboring villages in the eastern province of Saudi Arabia	90
Bahrain	Rajab et al. (2006) [33]	Sickle cell disease + *β*-thalassemia	Mean (SD) = 27.6 (5.2)	No	No	Yes	International Journal of Gynaecology and Obstetrics	Five years	Retrospective case-control study	Pregnant women delivered at Salmaniya Medical Complex and affiliated hospitals in Bahrain, who were mostly from Shia villages	351
Qatar	Fawzi et al. (2003) [47]	Sickle cell disease + *β*-thalassemia	NA	No	No	Yes	Qatar Medical Journal	78 months	Retrospective study	Hemoglobin Electrophoresis of patients to investigate for thalassemia or hemoglobinopathies by hematology laboratory of Hamad Hospital from January 1994 to June 2000	3275

**Table 2:** Bias assessment according to the Newcastle–Ottawa Quality Assessment Scale criteria.

Country	Study ID	Selection				Comparability	Outcome				Quality score
		Representativeness of exposed cohort	Selection of the non-exposed cohort from the same source as the exposed	Ascertainment of exposure	The outcome of interest was not presented at the start of the study	Comparability of cohorts	Assessment of outcome	Follow-up period long enough for an outcome to occur (median more than six months)	Adequacy of follow-up	Statistical test	
Patients with thalassemia											
Saudi Arabia	AlHamdan et al. (2007) [41]	Yes	Unclear	Yes (all screened individuals informed about the procedure)	Yes	Unclear	By clinicians	Unclear	Unclear	Unclear	Fair
	Alsaeed et al. (2011) [39]	Yes	Unclear	Yes (written informed consent obtained from all subjects)	Yes	Unclear	By clinicians	Unclear	Unclear	Available	Fair
	Alsaeed et al. (2018) [42]	Yes	Unclear	Yes (the data obtained in a deidentified format to protect the personal information of subjects)	Yes	Unclear	By clinicians	Unclear	Unclear	Available	Fair
	Ankra-badu et al. (2001) [43]	Yes	Unclear	Unclear	Yes	Unclear	By clinicians	Unclear		Yes	Unclear
	El-Hazmi et al. (1982) [44]	Yes	Unclear	Unclear	Yes	Unclear	By clinicians	Unclear		Yes	Unclear
	Memish et al. (2011) [45]	Yes	Unclear	Unclear	Yes	Unclear	By clinicians	Unclear	Unclear	Available	Fair
	Nasserullah et al. (2003) [46]	No	Unclear	Unclear	Yes	Unclear	By clinicians	Unclear	Unclear	Not available	Poor
	Ganeshaguru et al. (1987) [32]	No	Unclear	Unclear	Yes	Unclear	By clinicians	Unclear	Not available	Not available	Unclear
United Arab Emirates (UAE)	Barakat-Haddad et al. (2013) [36]	Yes	Unclear	Yes (the UAE ministry of education confirmed this study)	Yes	Unclear	Self-reported	Unclear	Unclear	Available	Good
	Denic et al. (2013) [37]	Yes	Unclear	Yes (written consent obtained)	Yes	Unclear	By clinicians	Unclear	Unclear	Available	Fair
	El-Kalla et al. (1998) [38]	Yes	Unclear	Yes (written consent obtained)	Yes	Unclear	By clinicians	Unclear	Unclear	Not available	Fair
Kingdom of Bahrain	Al-arrayed et al. (2003) [34]	Yes	Unclear	Yes (81% of parents' consent on screening)	Yes	Unclear	By clinicians	Unclear	Unclear	Unclear	Fair
Kuwait	Adekile et al. (1996) [35]	Yes	Unclear	Yes (written consent was obtained from the parents)	Yes	Unclear	By clinicians	Yes	Yes	Unclear	Good
Patients with *β*-thalassemia and sickle cell disease											
Saudi Arabia	Pembrey et al. (1980) [30]	Yes	Unclear	Yes	Yes	Unclear	By clinicians	Unclear	Unclear	Not available	Fair
	Perrine et al. (1981) [31]	Yes	Unclear	Unclear	Yes	Unclear	By clinicians	Unclear	Unclear	Available	Fair
	El-Hazmi et al. (1985) [40]	Yes	Unclear	Unclear	Yes	Unclear	By clinicians	Unclear	Unclear	Not available	Poor
Bahrain	Rajab et al. (2006) [33]	Yes	Yes	Unclear	Yes	Yes	By clinicians	Unclear	Unclear	Available	Fair
Qatar	Fawzi et al. (2003) [47]	No	Unclear	Unclear	Yes	Yes	By clinicians	Yes	Yes	Not available	Fair

**Table 3:** Prevalence of thalassemia in GCC countries.

Country	Study ID	Children below five years	Children above five years	Adults	Event	Total	Percent
Patients with thalassemia							
Saudi Arabia	AlHamdan et al. (2007) [41]	No	No	Yes	348	488315	0.07%
	Alsaeed et al. (2011) [39]	No	No	Yes	33	329	10.0%
	Alsaeed et al. (2018) [42]	No	Yes	Yes	787	1230582	0.064%
	El-Hazmi et al. (1982) [44]	Yes	Yes	Yes	Not mentioned	2643	30.72%
	Memish et al. (2011) [45]	No	No	Yes	29 006	1572140	1.8%
	Nasserullah et al. (2003) [46]	Yes	No	No	7923	21858	36%
	Ganeshaguru et al. (1987) [32]	No	No	Yes		840	
	(i) *β*-thalassemia				25	840	3.0%
	(ii) *α*/*β*-thalassemia				8		0.9%
	(iii) *α*-thalassemia				364		43.3%
UAE	Barakat-Haddad et al. (2013) [36]	No	Yes	No	57	6329	0.9%
	Denic et al. (2013) [37]	No	No	Yes	2	5672	0.035%
	El-kalla et al. (1998) [38]	Yes	No	No	46	418	11%
Kingdom of Bahrain	Al-arrayed et al. (2003) [34]	No	Yes	Yes	5	5685	0.09%
Kuwait	Adekile et al. (1996) [35]	Yes	Yes	No	12	39	30.7%
Patients with *β*-thalassemia and sickle cell disease							
Saudi Arabia	Alsaeed et al. (2011) [39]	Yes	Yes	Yes	11	329	3.3%
	Pembrey et al. (1980) [30]	Yes	Yes	No	16	140	11.43%
	Perrine et al. (1981) [31]	Yes	No	No	6	2341	0.25%
Bahrain	Rajab et al. (2006) [33]	No	No	Yes	34	282	12%
Qatar	Fawzi et al. (2003) [47]	No	No	Yes	Not mentioned	3275	1.37%

## Data Availability

Data are available with the corresponding author.

## Abbreviations

GCC: Gulf Cooperation Council
NOQAS: Newcastle–Ottawa Quality Assessment Scale
UAE: United Arab Emirates.

## Supplementary Materials

Supplementary Materials Searching terms used in this study.
