# Supplementary material for: Epidemiology of Thalassemia in Gulf Cooperation Council Countries: A Systematic Review
Source: Biomed Res Int. 2020 Oct 28;2020:1509501. doi: 10.1155/2020/1509501 (PMC7644312; doi:10.1155/2020/1509501)
Supplement: Supplementary Materials — Searching terms used in this study. [file 1509501.f1.docx]

(thalassemia or Alpha (α)-thalassemia or Beta (β)-thalassemia or Sickle Cell OR Sickle Cell Anemias OR Sickle Cell Diseases OR sickle cell disease OR Sickle Cell Disorders OR Sickle Cell Disorders OR sickle cell anemia OR Anemia, Sickle Cell OR Sickling Disorder Due to Hemoglobin S sickle cell trait OR Cell Trait, Sickle OR Cell Traits, Sickle OR Sickle Cell Traits OR Trait, Sickle Cell OR Traits, Sickle Cell OR Disease, Hemoglobin SC OR Diseases, Hemoglobin SC OR Hemoglobin SC Disease OR Hemoglobin SC Diseases OR HbS Disease OR Hemoglobin SC Disease OR SC Disease OR SC Disease, Hemoglobin OR SC Diseases, Hemoglobin OR Sickle Cell Hemoglobin C Disease OR SC Disease OR Disease, SC OR Diseases, SC OR SC Diseases) AND (epidemics OR Risk Factor OR Risk Factors OR Factor, Risk OR Factors, Risk OR Population at Risk OR Risk, Population at OR Populations at Risk OR Risk, Populations at OR etiology OR associated disease OR coexistent conditions OR concomitant conditions OR concomitant disease OR associated conditions OR coexistent disease OR causality OR causes OR pathogenesis OR prognosis OR Prognoses OR Prognostic Factors OR Factor, Prognostic OR Factors, Prognostic OR Prognostic Factor OR sequels OR frequency OR surveillance OR morbidity OR occurrence OR outbreaks OR prevalence OR endemics OR mortality OR incidence OR epidemiology OR complications AND (GCC countries OR Gulf Corporation Council countries OR Arabian gulf countries OR AGCC OR Bahrain OR Kuwait OR Oman OR Qatar OR Saudi Arabia OR UAE OR United Arab Emirates OR SA)
